# 

**DOI:** 10.1192/bjb.2023.11

**Published:** 2024-02

**Authors:** Martin Curtice

**Affiliations:** is a consultant in old age psychiatry with Coventry and Warwickshire Partnership NHS Trust, based at St Michael's Hospital, Warwick, UK. Email: mjrc68@doctors.org.uk

**Figure:**
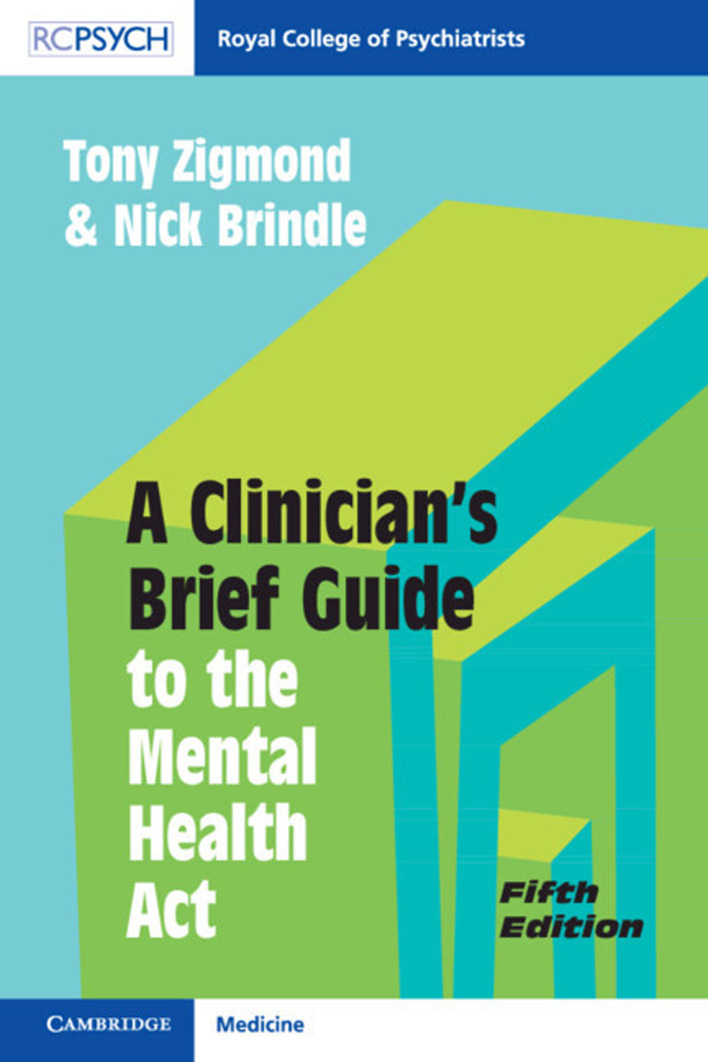

The fifth edition of this book very much holds firm to its stated aim in the preface to the first edition (2011) – it remains a ‘how to’ book designed as an ‘easy-to-read’ and interesting guide to the application of the Mental Health Act (MHA) in clinical practice in England and Wales (but also reviews important related statutes with which it interacts, including the Mental Capacity Act 2005 (MCA), Deprivation of Liberty Safeguards and Human Rights Act 1998). Although a brief guide, it remains comprehensive in its coverage. Case law and legal discourse can be dry at times, but this is bypassed by the authors using a conversational and flowing style of writing. They readily get to the crux of issues and explain them. Helpful note boxes punctuate the text throughout to highlight key points and provide sage practical advice.

The book starts by setting the MHA scene with a whistle-stop, but interesting tour of the evolution of law in the UK in relation to current mental health statutes. Another chapter clarifies the terminology and remit of the more commonly used civil sections and provides excellent advice about the practicalities and pragmatics of MHA assessments, for example assessment in a patient's home. Similar practical advice includes the completion of MHA forms. There is good explanation of how to address the MHA and MCA interface, which can be a vexing problem.

The chapter on community treatment orders (CTOs) elucidates issues around implementation and advice when attaching CTO conditions. Plentiful practical advice is found in the chapter about tribunals and hospital managers’ hearings, for example advice on report writing and verbal evidence. It also clarifies capacity aspects of patients being able to appeal their section and being able to participate in a hearing, reflecting recent case law development. A core chapter reviews consent to treatment. The authors note ‘This isn't an easy topic’ as it often involves overlapping statute and the juxtaposition of both mental and physical disorders. There is an enlightening chapter on medical treatment for physical disorders that co-occur with mental disorders and may or may not be related. The authors explore a legal problem in the use of section 58, and although they don't have an answer, they do provide guidance. This same chapter includes a lot of practical guidance on prescribing under the MHA, including common pitfalls to look out for.

This book is a must for trainees, being ideal to help learn about the intricacies and practicalities of the MHA. It is also an excellent refresher text for more experienced clinicians (and even to have at hand for MHA assessments, as the authors suggest). An overdue and major reform of the MHA after 40 years is on the near horizon, but this publication will hopefully continue with updates until then, and thereafter morph into a guide for any new MHA, owing to its niche importance in this area.

